# Primary Oral Health Care in India: Vision or Dream?

**DOI:** 10.5005/jp-journals-10005-1369

**Published:** 2016-09-27

**Authors:** Kotumachagi S Suresh, Pravin Kumar, Nagarathna Javanaiah, Shruti Shantappa, Pooja Srivastava

**Affiliations:** 1 Professor and Head, Department of Pedodontics, Government Dental College and Research Institute, Bengaluru, Karnataka, India; 2 Postgraduate, Department of Pedodontics and Preventive Dentistry Government Dental College and Research Institute, Bengaluru Karnataka, India; 3 Lecturer and Assistant Professor, Department of Pedodontics, Government Dental College and Research Institute, Bengaluru, Karnataka, India; 4 Postgraduate Student, Department of Pedodontics, Government Dental College and Research Institute, Bengaluru, Karnataka, India; 5 Postgraduate Student, Department of Pedodontics, Government Dental College and Research Institute, Bengaluru, Karnataka, India

**Keywords:** Dental home, Early childhood caries, Primary prevention.

## Abstract

The contemporary approach to dental caries management in children focuses on prevention than treatment. Pediatricians, general dentists and pediatric dentists must be involved in a detailed preventive program, which includes prenatal counselling, treatment of expectant mothers at risk for dental caries, infant oral health care and the establishment of the dental home, so that dental disease can be prevented in infants, starting at a young age.

Various health care system and organizations in India must join together to promote oral health care for all the children and specially focused toward children from disadvantaged background and children with special health care needs.

**How to cite this article:**

Suresh KS, Kumar P, Javanaiah N, Shantappa S, Srivastava P. Primary Oral Health Care in India: Vision or Dream? Int J Clin Pediatr Dent 2016;9(3):228-232.

## INTRODUCTION

Dental caries is one of the most common chronic diseases of early childhood.^1^ Oral diseases represent “what amounts to a silent epidemic affecting our most vulnerable citizens - poor children.” Early childhood caries (ECC) is one such form of dental caries that affects teeth of infants as soon as they erupt.^2^ Early childhood caries prevalence varies from population to population, but children of disadvantaged populations have been found to be most vulnerable.^3^ The prevalence of ECC in these developing countries is reported to be as high as 70%.^4^ Some of the studies reflect the significantly high burden of ECC among the children of age under five in India.^5^ To prevent caries in children, high-risk individuals must be identified at an early age (preferable low socioeconomic expected mothers during prenatal care), and aggressive strategies should be adopted, including anticipatory guidance, behavior modifications (oral hygiene and feeding practices), and establishment of dental home by 1st birthday for children at high risk.

An expected mother’s oral health throughout pregnancy will help establish a solid foundation to promote good oral health for both child and mother after birth. Accordingly, mothers with poor oral health and high levels of bacteria before and during pregnancy are at greater risk for delivering preterm, low-birth-weight babies;^6^ further, these children also have a greater risk for developing oral infections and dental caries.^7^ If the right preventive measures are taken early on, it is possible for parents to raise a cavity-free child. Since most dental diseases are nearly 100% preventable, if appropriate preventive measures are applied early enough, it may be possible to prevent dental caries.

Preventive measures can be divided into various stages.

### Stage 1 - Expectant Mothers/ Prospective Parents

Untreated dental caries in mothers increases the risk of caries development among their children, as maternal transmission and ECC have been established.^8^ The vertical colonization of *Streptococcus mutans* from mother to infant is well documented.^9,10^ Studies have shown that initially these Streptococci are acquired by the children through their mothers around 2 years of age, which is the window of infectivity.^11^ In fact, genotypes of *S. mutans* in infants appear identical to those that present in mothers in approximately 71% of mother-infant pairs.^12^ High caries rates run in families,^13^ and are passed from mother to child from generation to generation. The children of mothers with high caries rates are at a higher risk of decay.^14^ Therefore, decreasing the maternal cariogenic bacterial load is vital for the prevention or delaying of the infant developing ECC.^8^ So, it is important to meet the expectant mothers/prospective parents at an early stage. Gynecologists, family physician, auxiliary nurse midwife (ANM), Accredited Social Health Activists (ASHAs) under National Rural Health Mission (NRHM) are the people who come in contact with them much before a dentist. Dental surgeon must establish communication with them such that effective and timely referral are made to dental outpatient department of a nearby government/ private hospital.

In general, government hospitals (GH) provide services to people from low socioeconomic status. Individuals from lower economic strata experience financial, social, and material disadvantages that compromise their ability to care for themselves, obtain professional oral health care services, all of which lead to reduced resistance to oral and other diseases.^15^ Government should frame health care policies keeping in mind that oral health is no longer a neglected entity of general public health. Severe ECC can lead to malnourishment.^16^ Indian statistics have showed that 50.3% boys and 47.4% girls below 5 years were malnourished and underweight.^17^ Early childhood caries can be considered as one of the contributing factors for this situation. Studies also have shown that ECC can be a risk marker for anemia and problems associated with growth.^18^ Hence, it is important to identify the risk factors of ECC and prevent rather than merely treat it. Treating ECC could be an expensive issue and may place an unbearable burden on Indian economy.

In this regard prenatal counseling generally can be provided in conjunction with programs conducted in GH or primary health centers (PHCs). A close collaboration among members of various health professionals and community support groups (e.g., dentist, physicians, nurses, ANM, ASHAs, nutritionists, social workers) are important to ensure appropriate scheduling of presentations and reinforcement of concepts. A notice, such as “Want to benefit your child teeth before child-birth -visit Dental OPD” can attract the attention of expectant parents if displayed in hospitals in front of general OPD and pregnancy scanning room.

The counseling should provide participants with information about the development of oral structures and functions, dental disease processes, and recommended preventive measures (such as brush their teeth thoroughly twice a day with fluoridated toothpaste and spit out the excess after brushing, should rinse every night with an alcohol-free fluoridated mouth rinse, and to floss daily). In addition, the program should provide information on the importance of the mother’s diet, health-related behaviors during pregnancy (including the effects of drugs and tobacco), pregnancy gingivitis, and recommended scheduling of dental visit (preferably second trimester) for examination and restoration of all active decay as soon as feasible.

### Stage 2 - Infancy (0-1 Year)

If appropriate measures are applied early enough, it may be possible to totally prevent oral disease. The American Academy of Pediatric Dentistry (AAPD) recommends that infants (and parents) be scheduled for an initial oral evaluation visit within 6 months of the eruption of the first primary tooth but by no later than 12 months of age.^19^ Initial dental appointment at around 12 months of age is to assess the infant’s risk for dental disease, completion of clinical examination, provision of anticipatory guidance information, and a follow-up appointment.^20^

During postnatal counseling, information regarding teething problems and its management should be mentioned because, most likely it will be the first oral issue that parents confront. The pediatrician should advice mothers to breast feed the infant for approximately the first 6 to 12 months of life. The new mother should be motivated to clean the infant’s gum with a clean damp cloth or an infant toothbrush with a small head using plain water after each feeding. To prevent the transmission of bacteria that cause tooth decay via saliva from mother to child, parents should not share utensils with the child. The infant should never be put to sleep with a bottle or sippy cup in the mouth, and never allowed frequent or prolonged feeds with beverages high in sugar. Never dip a pacifier into honey or anything sweet before giving it to a baby. If an infant falls asleep while feeding, the teeth should be cleaned before placing the child in bed. Pediatricians should tell parents to hold infant while feeding and never to prop the bottle using pillows or other objects to hold the bottle. This propping can injure the unattended infant, and prolonged propping and feeding can cause ECC. Some parents also add sugar to a bottle of the infant but pediatricians and dentists should discourage this practice because it will cause sugary foods to pool around teeth increasing the risk for decay. Parents can introduce a small cup when the infant can sit up without support, and the infant should be weaned from the bottle when she/he starts to eat more solid foods and made to drink from a cup. Parents should not introduce juice into an infant’s diet before age 6 months and limit it to 4 to 6 oz per day. Infants of 6 months and older should receive age-appropriate healthy foods as recommended by their pediatrician and avoid snacking between meals. Foods containing sugar should be served to infants at mealtimes only and in limited amounts. Pediatricians should have the knowledge or be able to do the required referral for good nutrition and dietary counseling to support oral and general health of the child.^21^

An oral examination of an infant or toddler is usually accomplished through the help of a parent. The knee to knee position allows for the parent to help restrain a child, and provides dentist as well as the parent with good vision of the child’s oral cavity. Following the examination and prophylaxis, preventive recommendations should be formalized based on the risk assessment factors, family and health history, and results of the oral examination. Anticipatory guidance, a guide on what to expect as the child enters the next developmental stage, can be provided to the parent and family.^22^

Dental Home Concept

The AAPD first issued its support of the dental home concept in 2001 after evaluating the success of the medical home policy put forth by the American Academy of Pediatrics in 1992.^23^ The dental home model follows many of the same principles of the patient-centered medical home model. There is strong clinical evidence for the efficacy of dental care early on in a child’s life coupled with caries-risk assessment, anticipatory guidance, and periodic supervision.^24^ It has been demonstrated that children who have a dental home are more likely to receive preventive and routine oral health care.^23^

The definition states:

“The dental home is the ongoing relationship between the dentist and the patient, inclusive of all aspects of oral health care delivered in a comprehensive, continuously accessible, coordinated, and family-centered way. Establishment of a dental home begins no later than 12 months of age and includes referral to dental specialists when appropriate.”^25^

Services provided by dental home:^26^

- Schedule early dental visits at approximately 12 to 18 months of age.
- Assess the risk of the infant and toddler for future dental disease.
- Evaluate the fluoride status of the infant and make appropriate recommendations.
- Demonstrate to caretakers the appropriate method for cleaning the tooth.
- Discuss the advantage/disadvantage s of non-nutritive sucking.
- Be prepared to treat the infant/toddler if ECC is diagnosed or to make the appropriative referral.
- Be available 24 hours a day, 7 days a week to deal with any acute dental problems.
- Recognize the need for specialty consultation and referrals.^26^

However, the total numbers of dentists are inadequate to provide a dental home for the total numbers of children, so priority should be given to children at greater risk for dental disease, including those with earliest signs of ECC, children from high-risk subpopulations, and children with special health care needs.^25^

Health and family welfare department of India do not have enough trained clinicians to staff this model of care. Retooling practices and training clinicians to deliver team-based care will require substantial short- and long-term financial resources. Coordination of physicians, dentists, and paramedical staff is needed to deliver team-based care in government/private hospitals and use of health information technology to improve care, and apply evidence-based principles in practice. However, current training programs do not educate young physicians, dentists, and paramedical staff in the fundamental precepts of the Dental home concept.

### Stage 3 - Care for Younger Children

Oral hygiene measures must be implemented as soon as the first tooth erupt into the oral cavity. The oral health care measures include the following:^21,25-28^

- Limit the frequency of snacking, which can increase a child’s risk of developing caries.
- Parents should use a “smear” of toothpaste to brush the teeth of a child less than 2 years of age and perform or assist with their child’s tooth brushing. Unless advised to do so by a dentist or other health care professionals, parents should not use fluoride toothpaste for children less than 2 years of age.
- For the 2 to 5 years old child, parents should dispense a “pea-size” amount of toothpaste and perform or assist with their child’s tooth brushing. Children should be taught to never swallow the toothpaste.
- Dental flossing should be initiated when adjacent tooth surfaces cannot be cleansed by a toothbrush.
- Brushing should be supervised and assisted until 7 and 8 years of age (an age at which they can tie their own shoe laces). A small, circular scrubbing motion is recommended for children.
- Tropical fluoride (like varnish, gels, foams) applications may be especially effective in children with a high-risk of tooth decay.
- Application of dental sealants based on caries risk assessment, creates a physical barrier against dental plaque and this barrier prevents tooth decay.
- Thumb sucking is normal for infants; most stop by age of 2 and it should be discouraged after age 4. Prolonged thumb sucking can create crowded, crooked teeth, or bite problems. Dentists can suggest ways to address a prolonged thumb sucking habit.
- Parents and caregivers need to take care of their own teeth so that cavity-causing bacteria are not as easily transmitted to children.

## CHILDREN WITH SPECIAL HEALTH CARE NEEDS

Infants and children with special health care needs may be at increased risk for developing oral conditions like delayed tooth eruption, malocclusion, dental caries, dental anomalies, trauma, infections, and gingival enlargements. All of these conditions are attributed to several congenital syndromes, medications, or inherent immune deficiencies and include Down’s syndrome, Treacher-Collin’s syndrome, and ectodermal dysplasia. Several medications cause gingival enlargements; for example, dilantin (phenytoin sodium) and phenobarbital, which are prescribed for epilepsy, can cause gingival hyperplasia. It is very important for a general health professional to be aware of these conditions: Children with these conditions may need dental referrals on a regular basis. Like all children, these children should have their first visit within 6 months of eruption of the first tooth or at 12 months of age. However, future visits may have to be more frequent. Dental treatments may require additional time to accommodate the child’s condition, medications, behavior, and complexity of care. Most important, these children should receive oral health care from those who have experience with this population.^21^

Preventive services include the following^29^:

- Initial and periodic examinations of the dentition and oral cavity, including medical and dental histories, furnished in accordance with the attached periodicity schedule.^30^
- Education for the patient and the patient’s family on measures that promote oral health as part of initial and periodic child assessment.
- Age-appropriate anticipatory guidance and counseling on non-nutritive habits, injury prevention, and tobacco use/substance abuse.
- Application of topical fluoride at a frequency based upon caries risk assessment.
- Prescription of dietary fluoride supplement^31^ based upon a child’s age, caries risk, and fluoride level of the water supply.
- Application of pit and fissure sealants based on caries risk assessment, not based on patient age or time lapsed since eruption.^32^
- Oral prophylactic services at a frequency based on caries and periodontal risk factors.
- Challenges for implementing Primary Oral Health Care in India.

Traditionally, medical and dental care have been two separate streams of the health care services in the Indian Medical system. The current training programs in the health care field presents an unprecedented opportunity for the integration of medical and dental care. Medical and dental training programs need to develop innovative training models that reflect evolving models of health care delivery, such as the primary oral health care.

Data indicate that children from low-income households are more likely to experience caries and have higher levels of untreated caries compared with their more economically advantaged counterparts.^33^ Early childhood dental caries is seen in epidemic proportions and more prevalent in children in families belong to the low-income group.^25^ The government hospital are the one which serve people of low socioeconomic status in large scale. To prevent ECC in children, high-risk individuals must be identified at an early age (preferably high-risk mother during pregnancy) and oral health counseling should be given. In order to give prenatal counseling the dentist/ allied health care professionals should work arm in arm with gynecologists, pregnancy scanning specialist, nurses, ANM, ASHAs, and all other health care professionals. Inform obstetricians and pediatricians to refer expectant mothers to dental OPD in order to receive prenatal counseling and modification of mother’s dental flora which can have a significant impact on the child caries rate.^34-36^

To institute the primary oral health care in our country, a transformation must occur in which physicians and dentist who practice independently with minimal staff support in government sector or use of information technology will need to learn to adopt the primary oral health care model. Medical and dental education department should include training programs with coordinated emphasize health care systems for health of population in a continuous and preventive care.

Our recommendations for effective implementation of primary oral health care in India are:

- Compulsory dental counseling for beneficiaries of Janani Sureksha Yojana scheme.
- Include dental examination/fitness form in preschool admission forms.
- Compulsory postings during internship in orphanage; home for mentally and physically disabled children.
- Government-aided oral health camps for children with special health care needs.
